# Incorporation of native antibodies and Fc-fusion proteins on DNA nanostructures *via* a modular conjugation strategy†
†Electronic supplementary information (ESI) available: Experimental methods, DNA origami design, DNA sequences, and additional experimental data. See DOI: 10.1039/c7cc04178k


**DOI:** 10.1039/c7cc04178k

**Published:** 2017-06-09

**Authors:** Bas J. H. M. Rosier, Glenn A. O. Cremers, Wouter Engelen, Maarten Merkx, Luc Brunsveld, Tom F. A. de Greef

**Affiliations:** a Laboratory of Chemical Biology and Institute for Complex Molecular Systems, Eindhoven University of Technology, P.O. Box 513 , 5600 MB Eindhoven , The Netherlands; b Computational Biology Group, Department of Biomedical Engineering, Eindhoven University of Technology, P.O. Box 513 , 5600 MB Eindhoven , The Netherlands . Email: t.f.a.d.greef@tue.nl

## Abstract

A photocrosslinkable protein G variant was used as an adapter protein to covalently and site-specifically conjugate an antibody and an Fc-fusion protein to an oligonucleotide. This modular approach enables straightforward decoration of DNA nanostructures with complex native proteins while retaining their innate binding affinity, allowing precise control over the nanoscale spatial organization of such proteins for *in vitro* and *in vivo* biomedical applications.

## Abstract 2

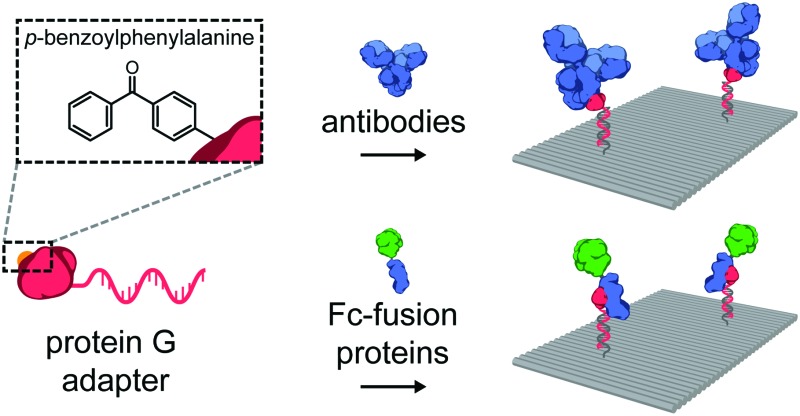
A photocrosslinkable protein G adapter was used to site-specifically conjugate complex native proteins to oligonucleotides, allowing for efficient incorporation on DNA origami nanostructures.

## 

The specificity and programmability of nucleic acid base-pairing is applied in the field of DNA and RNA nanotechnology to construct well-defined assemblies of molecules and other functional components.^1^–^4^ The DNA origami technique^5^ especially has been used extensively to create highly consistent nanoscale scaffolds for the organization of *e.g.* peptides, proteins, nucleic acids, polymers and nanoparticles.^6^ A key strength of DNA nanotechnology is the ability to precisely organize biomolecules on the order of 10–100 nm, a scale that is generally difficult to access using traditional biochemical approaches or top-down engineering.^7^ Proteins in particular are attractive targets, often operating in the cell in complex multi-component systems and networks, where multivalency and nanoscale spatial organization play an essential role.^8^–^12^ This has led to DNA and RNA nanostructures being employed as powerful tools to study enzymatic cascades,^13^ receptor activation,^14^,^15^ and as *in vivo* delivery vehicles.^16^,^17^

For these applications, the synthesis of DNA–protein conjugates is essential and as a result, a wide variety of conjugation strategies are available.^18^ Functionalized oligonucleotides (ODNs) can be coupled using chemical handles already present in the protein, such as cysteines and lysines, but this usually results in non-specific conjugation, limited control over stoichiometry, and concurrent loss of function.^19^–^21^ Alternatively, site-specific conjugates can be synthesized using either non-covalent recognition elements such as biotin–streptavidin and histidine-Ni^2+^-NTA, or covalent approaches, *e.g.* by using large self-labeling protein domains like the SNAP-, CLIP- or Halo-tags, or by introducing bio-orthogonal non-natural amino acids. Additionally, elegant hybrid strategies have been introduced that combine a site-specific non-covalent interaction to template a subsequent covalent coupling.^22^,^23^ In most of these strategies genetic re-engineering of the protein of interest is required to introduce the necessary modifications. While this is a feasible option for simple proteins and proof-of-principle studies, it can be difficult for larger, more complex proteins, which are often expressed in non-bacterial hosts and can require extensive optimization.

An important class of such proteins are antibodies, which recognize a wide range of molecular targets with extraordinary specificity and affinity, and therefore represent an attractive target for various fundamental applications. Indeed, the combination of nanoscale addressability of DNA nanotechnology and the specificity of antibodies has been exploited for targeted induction of apoptosis,^17^ for immunodiagnostic applications,^24^ and as an *in vivo* imaging tool.^19^,^25^ However, despite their wide-spread commercial availability, applications in this field have been limited due to the challenging process of synthesizing well-defined functional DNA–antibody conjugates. We therefore sought to develop a modular, universal strategy to incorporate antibodies onto DNA nanostructures, allowing their use in many *in vitro* and *in vivo* biomedical applications. Recently, Hui *et al.* reported on the light-activated site-specific conjugation (LASIC) of native human antibodies to various small molecules utilizing the high-affinity binding of protein G to the constant Fc region of immunoglobulin G-type (IgG) antibodies.^26^ The authors showed that introduction of the unnatural amino acid *p*-benzoylphenylalanine (BPA) in the Fc-binding site allowed for specific covalent conjugation through the photoreactive benzophenone moiety, without affecting the antigen-binding affinity. Since protein G can be straightforwardly expressed in *E. coli*, we envisioned that it could also be employed as a versatile adapter protein in a conjugation strategy for ODNs to native antibodies (Fig. 1A).

**Fig. 1:**
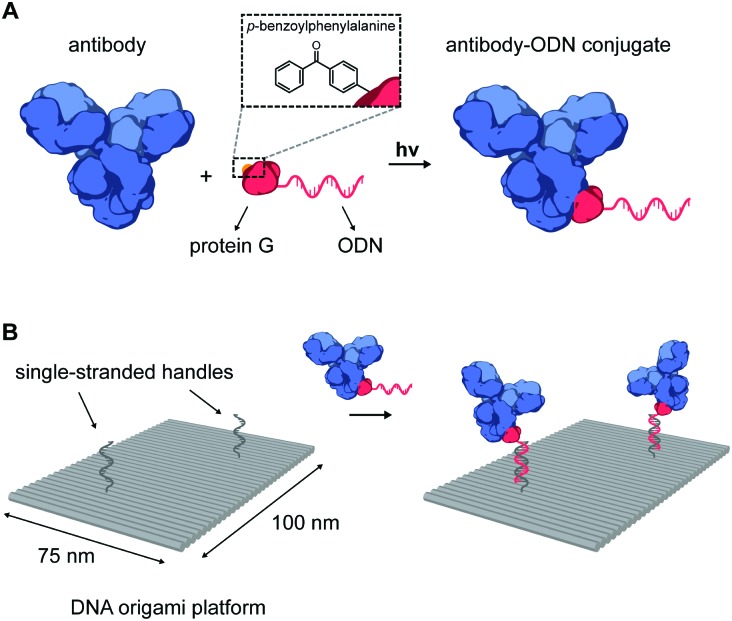
(A) Schematic overview of the site-specific conjugation strategy of a native antibody to an oligonucleotide (ODN) using a photocrosslinkable protein G adapter. The protein G variant is expressed carrying an N-terminal cysteine for coupling to a 20 nt 3′-amino-functionalized ODN using the heterobifunctional linker Sulfo-SMCC. Additionally, the non-natural amino acid *p*-benzoylphenylalanine (BPA) is incorporated into the Fc-binding domain of protein G to allow covalent crosslinking to the immunoglobulin G-type (IgG) antibody using long-wavelength UV light. (B) Illustration of the incorporation of antibodies on DNA nanostructures. Two-dimensional 75 × 100 nm^2^ DNA origami rectangles are designed to carry two handles protruding from its surface. DNA hybridization of the antibody–ODN conjugates to the complementary handles leads to incorporation of antibodies onto the DNA origami nanostructure at the programmed positions.

In our approach, the protein G variant developed by Hui *et al.*^26^ was modified to include an N-terminal *Strep*-tag, a C-terminal hexahistidine tag and a single cysteine at the N-terminus (see ESI†). This 9.6 kDa protein (pG) was expressed in *E. coli* using amber codon suppression with an engineered orthogonal amino acyl tRNAse/tRNA pair from *M. janaschii*,^27^ allowing incorporation of BPA in the Fc-binding domain. After purification by Ni^2+^-affinity chromatography and *Strep*-tactin affinity chromatography, pG was obtained in high yield (14 mg L^–1^ culture, see ESI†). Conjugation of pG to a 20 nt, amino-functionalized ODN was performed using the heterobifunctional crosslinker sulfosuccinimidyl 4-(*N*-maleimidomethyl) cyclohexane-1-carboxylate (Sulfo-SMCC). The reaction afforded the pG–ODN conjugate in low yields (∼15%), due to the formation of an unreactive thiazolidine adduct during pG expression (see Fig. S1, ESI†). We expect that the yield of the conjugation reaction can be improved by introducing *e.g.* an additional amino acid before the cysteine at the N-terminus of pG, or by reversing adduct formation using methoxyamine.^28^,^29^ Nevertheless, pure pG–ODN was obtained after removal of unreacted pG and ODN, by consecutive anion-exchange and Ni^2+^-affinity chromatography, respectively (see Fig. S3, ESI†).

Photoconjugation of pG–ODN to antibodies was tested using cetuximab, a monoclonal IgG1 antibody used as a therapeutic epidermal growth factor receptor (EGFR) inhibitor. A 5-fold molar excess of pG–ODN was added to 0.4 μM cetuximab and the solution was incubated for 2 h at 4 °C. After binding of pG–ODN to cetuximab, the benzophenone moiety in pG is expected to crosslink preferentially to methionine residues in the Fc region upon irradiation.^30^ Indeed, analysis using polyacrylamide gel electrophoresis under reducing conditions (SDS-PAGE) showed >90% covalent coupling of pG–ODN to the heavy chain of cetuximab only upon illumination with low-energy UV light (Fig. 2A). The extent of coupling of pG–ODN is similar to conjugation of pG alone, indicating that the ODN does not influence binding of pG to the antibody. We note that IgG-type antibodies like cetuximab are composed of two identical heavy chains, resulting in conjugation of up to two pG–ODN molecules per antibody. If needed, mono-conjugated antibody–ODN can be obtained by *e.g.* purification of the reaction mixture using immunoprecipitation with protein G or protein A resins, as shown previously.^26^

**Fig. 2:**
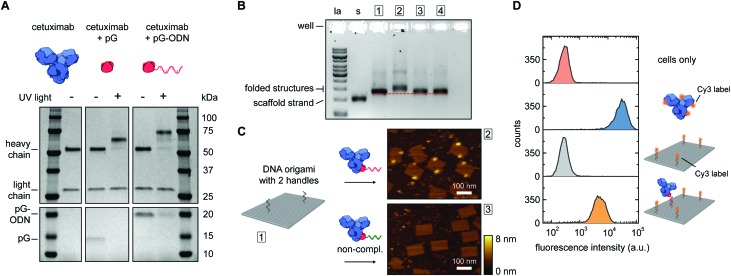
Synthesis and characterization of cetuximab-functionalized DNA nanostructures. (A) Antibody–ODN conjugation using the protein G adapter, analyzed with SDS-PAGE analysis under reducing conditions. Conjugation reactions were performed in 10 mM Tris, 1 mM EDTA, 100 mM NaCl, pH 7.5, with 0.4 μM of cetuximab and a 5-fold molar excess of protein G (pG, 9.5 kDa) or pG–ODN (16 kDa) for 2 h at 4 °C in the absence or presence of UV light (*λ* = 365 nm). (B and C) Characterization of cetuximab incorporation onto DNA nanostructures. Conjugation reactions were performed by combining 1 μM of pG–ODN with 5 equivalents of cetuximab for 2 h at 4 °C under UV light. DNA origami rectangles with 2 handles were folded and purified by spin filtration using standard protocols (see ESI†). Hybridization of cetuximab–ODN conjugates to the DNA nanostructures was done by incubating 8 nM DNA origami and 80 nM of cetuximab–ODN for 2 h at 4 °C. Subsequently, DNA nanostructures were purified using two rounds of PEG precipitation. (B) Gel-electrophoretic mobility of purified DNA nanostructures, assessed on a 1.5% agarose gel: (1) before incubation, and after incubation with (2) cetuximab–ODN conjugate, (3) conjugate with a non-complementary ODN, (4) cetuximab only. Labels: la, 1 kb ladder; s, single-stranded scaffold. (C) AFM height images of the purified DNA nanostructures, as listed in (B), showing successful incorporation of cetuximab–ODN on DNA nanostructures at programmed positions (2). Conjugates with a non-complementary ODN (3) do not show incorporation. (D) Flow cytometry analysis of cetuximab binding to EGFR-overexpressing human A431 carcinoma cells. Samples at a final cetuximab concentration of 1.75 nM were incubated with the cells for 30 min at room temperature in PBS supplemented with 0.1% (w/v) BSA (see ESI†). After washing, the fluorescence intensity of 5000 single cell events per sample was recorded, and the intensity distributions indicate EGFR-binding for both cetuximab and 1× cetuximab-functionalized DNA nanostructures. From top to bottom: cells only, cetuximab labeled with an average of 4 Cy3 labels, DNA origami rectangles with 4 Cy3 labels, and DNA origami rectangles with 4 Cy3 labels and 1 cetuximab.

The successful synthesis of antibody–ODN conjugates allows for incorporation of the antibody onto DNA nanostructures, using the ODN as an anti-handle for hybridization to a complementary single-stranded handle strand protruding from the surface of a DNA origami platform. As a model system, we used a two-dimensional 75 × 100 nm^2^ DNA origami rectangle with two handles on the surface at a distance of ∼40 nm (see Fig. 1B and Fig. S4, ESI†). After folding and purification using spin filtration, the DNA nanostructures were functionalized with cetuximab–ODN and purified using polyethylene glycol (PEG) precipitation (see Fig. S6, ESI†).^31^,^32^ An electrophoretic gel mobility shift assay comparing empty and functionalized DNA nanostructures indicated successful incorporation of cetuximab (compare lane 1 and 2, Fig. 2B). Control experiments, in which the anti-handle was not complementary to the handle (lane 3) or using only unconjugated cetuximab (lane 4), exhibited no gel shift.

To visualize cetuximab incorporation and confirm agarose gel results, atomic force microscopy (AFM) was used. Topographic AFM imaging under liquid conditions revealed well-folded DNA origami rectangles, with cetuximab present at the two programmed positions (Fig. 2C). Image analysis indicated an average incorporation efficiency of ∼70%, with approximately 50% of the DNA nanostructures functionalized with two antibodies (Fig. S7, ESI†). This is consistent with values found in literature,^33^,^34^ although incorporation efficiencies of up to 90% have been reported in literature for smaller proteins.^13^,^14^ Finally, flow cytometry was used to prove that cetuximab could still bind to its native target after incorporation on DNA nanostructures and subsequent purification. EGFR-overexpressing A431 carcinoma cells were incubated with either Cy3-labeled cetuximab or Cy3-labeled DNA origami functionalized with one cetuximab, and subjected to flow cytometry analysis. In both cases, an increase in mean fluorescence intensity of individual cells was observed, indicating binding of cetuximab to the EGFR receptor irrespective of the presence of the DNA nanostructures (Fig. 2D). Additionally, the binding strength of the interaction between EGFR and cetuximab was shown to be similar for both samples (see Fig. S5, ESI†) indicating that the antibody retains its affinity when bound to the DNA origami.

Thus far, we have shown ODN conjugation and DNA nanostructure functionalization of IgG antibodies, targeting their constant Fc domain *via* a protein G adapter. Interestingly, the Fc domain is often used as a fusion partner to biologically active proteins, increasing *in vivo* stability and circulation, and prolonging activity in therapeutic applications.^35^,^36^ As a result, a large library of growth factors, cell receptors, cell receptor ligands, cytokines, and other signaling proteins, are commercially available as Fc-fusion proteins.^37^ We hypothesized that this class of proteins should be fully compatible with our protein G-assisted conjugation strategy, and correspondingly, would allow modular incorporation of all Fc-fusion proteins onto DNA nanostructures (Fig. 3A).

**Fig. 3:**
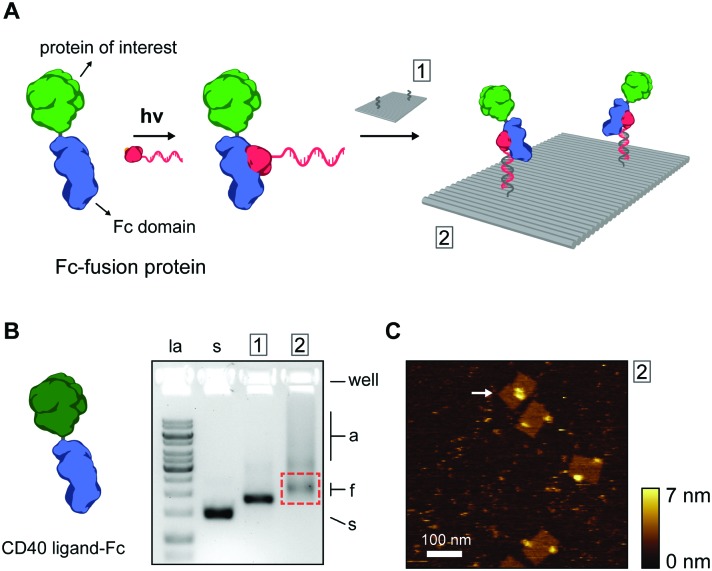
(A) Schematic overview of the incorporation of Fc-fusion proteins on DNA nanostructures. Fc-fusion proteins are composed of the constant Fc region of an IgG-type antibody fused directly to the protein of interest. After photoconjugation to pG–ODN, the Fc-fusion protein is incorporated onto the DNA nanostructure by hybridization with complementary handle strands. Note that Fc-fusion proteins are generally expressed as disulfide-bridged homodimers, but for simplicity only the monomer is depicted. (B) As a proof-of-principle, commercially available CD40 ligand Fc-fusion protein (CD40L, monomeric molecular weight, 43 kDa) was used. Gel-electrophoretic mobility of DNA nanostructures, (1) before, (2) and after functionalization with CD40L, assessed on a 1.5% agarose gel. Labels: la, 1 kb ladder; s, single-stranded scaffold; a, aggregated structures; f, correctly folded structures. Purification was done by agarose gel extraction from the region indicated by the red rectangle. (C) Corresponding AFM height image of the purified DNA nanostructures showing incorporation of CD40L at the two programmed positions. A possible intra-structural interaction of two CD40L is indicated by the arrow.

To test this, we used the model CD40 ligand-Fc fusion protein (CD40L), which is commercially available as a disulfide-bridged homodimer. The CD40 ligand is a transmembrane cytokine in active T cells and is critically involved in organizing and activating the CD40 cell receptor on antigen-presenting cells, leading to various downstream immune responses.^38^,^39^ Photoconjugation of CD40L to pG–ODN was performed as described for cetuximab and SDS-PAGE analysis confirmed synthesis of CD40L–ODN conjugates in >90% yield (see Fig. S8, ESI†). Incorporation of CD40L–ODN onto DNA nanostructures was performed using the DNA origami rectangle with two handles. Agarose gel electrophoresis showed that CD40L–ODN hybridization resulted in the expected gel mobility shift, but also in an increase in aggregation of the DNA origami structures (compare lane 1 and 2, Fig. 3B). We attribute this aggregation to the tendency of the CD40 ligand to form trimers in solution,^40^ effectively leading to DNA origami bridging by CD40L. Nevertheless, functionalized DNA nanostructures were isolated by gel extraction and subsequent AFM imaging confirmed well-formed DNA origami with proteins in the two programmed positions (Fig. 3C). In some occasions, a possible interaction between two CD40L proteins on the same DNA origami was observed (white arrow, Fig. 3C). Even though the designed distance between the two handle strands is 40 nm, the flexible nature of the CD40L–ODN complex and the DNA origami structure itself could give rise to such intra-structural protein interactions.

In conclusion, we have presented a modular strategy to covalently conjugate oligonucleotides to proteins containing an Fc domain using a versatile photoreactive protein G adapter. While most DNA–protein conjugation methods require laborious and challenging chemical modifications to the protein of interest, the current method can be applied to a large library of commercially-available proteins, including antibodies, growth factors, cell receptors, and cytokines. Incorporation of such complex proteins onto DNA origami platforms can lead to the development of powerful functional nanostructures to study, for example, the effects of receptor clustering in signal transduction, and other biomedical applications.

We thank Remco Arts and Benice van Gerven for initial photoconjugation experiments and for kindly providing both the pET28a-pG and pEVOL-pBpF plasmids, Anniek den Hamer and Berta Gumí Audenis for useful discussions, and the ICMS Animation Studio for help with preparing the figures. This work was supported by European Research Council (ERC) Starting Grants (BioCircuit 677313 and ERC-2011-StG 280255), funding from the Netherlands Organization for Scientific Research (NWO) through ECHO-STIP grant 717.013.001 and VICI grant 016.150.366, and funding from the Ministry of Education, Culture and Science (Gravity programme, 024.001.035).

## Supplementary Material

Supplementary information
