# Supplementary material for: Incorporation of native antibodies and Fc-fusion proteins on DNA nanostructures via a modular conjugation strategy
Source: Chem Commun (Camb). 2017 Jun 9;53(53):7393–6. doi: 10.1039/c7cc04178k (PMC5708335; doi:10.1039/c7cc04178k)
Supplement: Supplementary file 1 [file CC-053-C7CC04178K-s001.pdf]

Electronic Supplementary Material (ESI) for ChemComm.
This journal is © The Royal Society of Chemistry 2017

Incorporation of native antibodies and Fc-fusion proteins on DNA nanostructures via a modular conjugation strategy

Bas J.H.M. Rosier, ‡[a] Glenn A.O. Cremers, ‡[a] Wouter Engelen,[a] Maarten Merkx,[a] Luc Brunsveld[a] and Tom F.A. de Greef*[ab]

[a]Laboratory of Chemical Biology and Institute for Complex Molecular Systems, Department of Biomedical Engineering, Eindhoven University of Technology, The Netherlands.

[b]Computational Biology group, Department of Biomedical Engineering, Eindhoven University of Technology, The Netherlands.

*Corresponding author: t.f.a.d.greef@tue.nl.

‡ These authors contributed equally.

Electronic Supplementary Information

Contents

Expression and purification of the protein G adapter (pG) 2
DNA and protein sequence of pG 4
Synthesis and purification of protein G-oligonucleotide conjugates (pG-ODN) 4
Design of the DNA origami rectangle 6
Folding and purification of DNA origami nanostructures 8
Photoconjugation of cetuximab and incorporation onto DNA nanostructures 8
A431 cell culturing and analysis using flow cytometry 9
Photoconjugation of CD40L and incorporation onto DNA nanostructures 11
AFM imaging of DNA nanostructures 11
Table S1: mutagenesis primers 14
Table S2: handle and anti-handle sequences 14
Table S3: sequences of unmodified staple strands 14
Supplementary references 18

Expression and purification of the protein G adapter (pG)

The gene for pG was based on the sequence used by Hui *et al.*[1] and was available in a pET28 expression vector with an N-terminal *Strep*-tag, kindle provided by Remco Arts. In this construct, the native alanine at position 36 has been mutated to encode for an amber stop codon for incorporation of *p*-benzoylphenylalanine (BPA). A C-terminal hexahistidine tag was introduced via Liu PCR[2] using Phusion polymerase (New England Biolabs) and partially overlapping primers (see Table S1). An N-terminal cysteine was introduced using the QuikChange Lightning Multi Site-Directed Mutagenesis kit (Agilent), according to the manufacturer's instructions and using the primer in Table S1. The pEVOL-pBpF vector, encoding for the orthogonal aminoacyl tRNA synthetase/tRNA pair, was kindly provided by Peter Schultz (Addgene plasmid 31190). Both plasmids were co-transformed into *E. coli* BL21(DE3) competent bacteria (Novagen) and cultured at 37°C in 500 mL 2xYT medium (16 g/L peptone, 5 g/L NaCl, 10 g/L yeast extract) supplemented with 50 µg/mL kanamycin (Merck) and 25 µg/mL chloramphenicol (Sigma Aldrich). Protein expression was induced at OD_{600}=0.6 by addition of 1 mM β-D-1-thiogalactopyranoside (IPTG, Applichem) and 0.02% (w/v) arabinose (Sigma Aldrich). Simultaneously, the unnatural amino acid BPA (dissolved in 0.5 M NaOH, Bachem) was added to the culture medium at a final concentration of 1 mM. Expression was carried out for ~18 h at 25°C. Cells were harvested by centrifugation at 10,000 g for 10 min at 4°C and lysed by resuspending the pellet in BugBuster (5 mL/g pellet, Merck) supplemented with benzonase (5 µL/g pellet, Merck) for 45 min on a shaking table at room temperature. After centrifugation at 40,000 g for 30 min at 4°C the soluble fraction containing pG was collected.

Purification was performed by sequential Ni^{2+}-affinity chromatography and *Strep*-tactin affinity chromatography (Fig. S1a). The soluble fraction was first loaded on a Ni-charged column (His-Bind® Resin, Novagen) and washed with wash buffer (1x PBS, 370 mM NaCl, 10% (v/v) glycerol, 20 mM imidazole, pH 7.4). Protein was eluted with elution buffer (1x PBS, 370 mM NaCl, 10% (v/v) glycerol, 250 mM imidazole, pH 7.4) and directly applied to a *Strep*-tactin column (Superflow® resin, IBA Life Sciences). The column was washed with wash buffer (100 mM Tris-HCl, 150 mM NaCl, 1 mM EDTA, pH 8.0) and the protein eluted with wash buffer supplemented with 2.5 mM desthiobiotin (IBA Life Sciences). The concentration of pG was determined by measuring the absorption at 280 nm (ND-1000, Thermo Scientific) assuming an extinction coefficient of 15,470 M^{-1} cm^{-1}. Total yield after purification was ~14 mg/L culture medium. Purity of pG was assessed on 4-20% SDS-PAGE precast gels (Bio-Rad) under reducing conditions, stained with Coomassie Brilliant Blue G-250 (Bio-Rad). Proteins were snap frozen in liquid nitrogen and stored in 500 µL aliquots of 50 µM at -80°C in a buffer containing 100 mM Tris-HCl, 150 mM NaCl, 1 mM EDTA and 2 mM TCEP at pH 8.0.

Molecular weight was confirmed using liquid chromatography quadrupole time-of-flight mass spectrometry (Fig S1b-d). An aliquot of pG was buffer exchanged to ultrapure H_2O (Merck Millipore) in Amicon 3 kDa MWCO centrifugal filters (Merck Millipore) to a final concentration of 1 mg/mL. A 0.1 µL sample was injected into an Agilent Polaris C18A RP column with a flow of 0.3 mL/min and a 15-60% acetonitrile gradient containing 0.1% formic acid. Mass spectra were measured on a Xevo G2 QTof mass spectrometer (Waters) in positive mode. Deconvolution of the m/z spectra was performed with MaxEnt Deconvolution software.

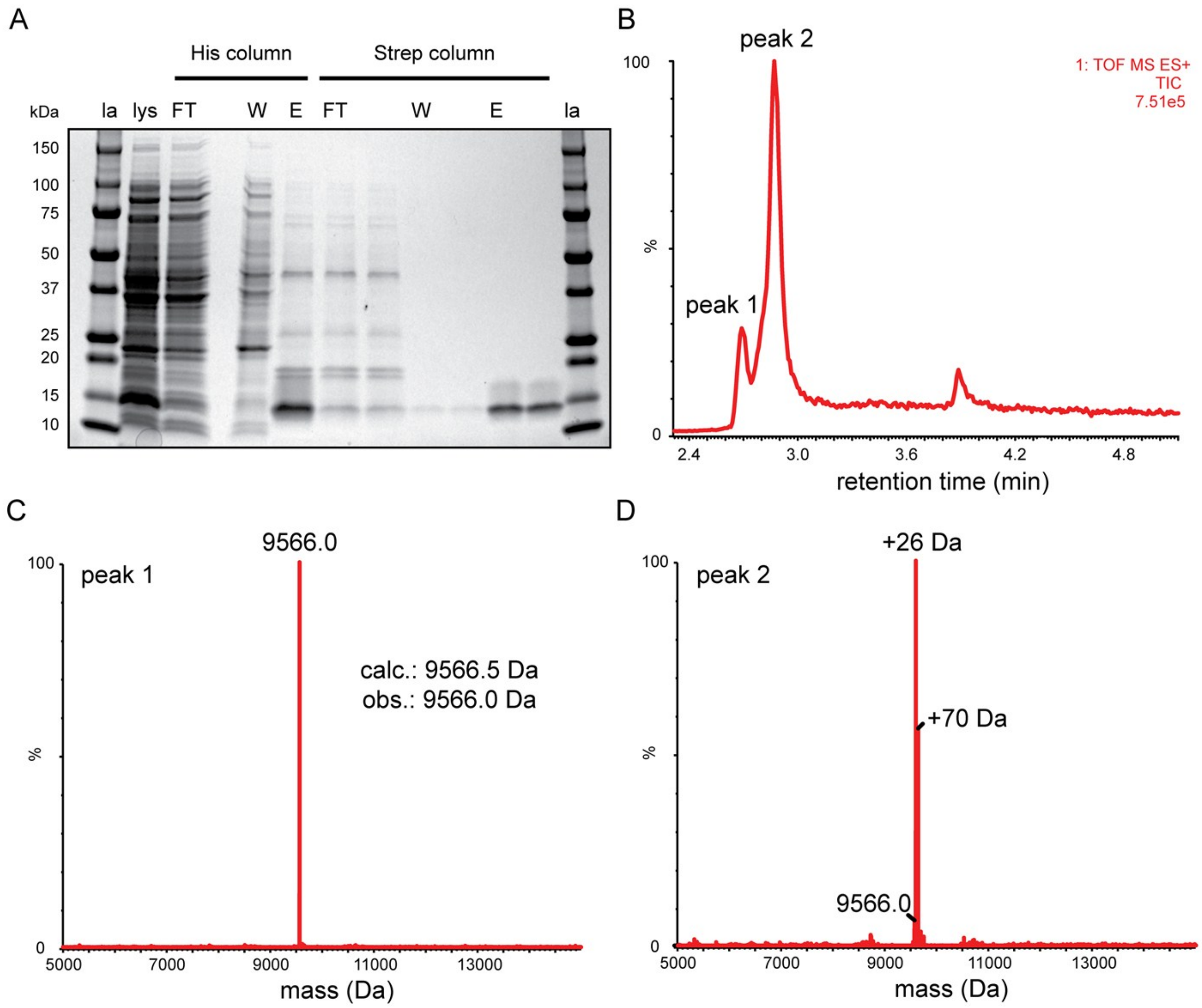

Figure S1 Purification and characterization of pG. (a) SDS-PAGE gel of pG expression and purification. Labels: la, ladder; lys, soluble fraction of cell lysate; FT, flow through fractions; W, wash fractions; E, elution fractions. On gel, pG has an apparent mass of ~14 kDa (calculated mass 9566.5 Da). (b) Liquid chromatography trace of the elution fraction showing two main peaks. (c) Deconvoluted mass spectrum of peak 1 shows a single peak corresponding to the calculated mass of pG without N-terminal methionine (9566.5 Da). (d) Deconvoluted mass spectrum of peak 2 shows a small peak corresponding to the calculated mass of pG and two adducts (+70 Da and +26 Da), which correspond to an unreactive thiazolidine adduct of pG at the N-terminal cysteine and a thiazolidine adduct after decarboxylation, respectively.[9]

DNA and protein sequence of pG

The single-letter amino acid code is shown in uppercase, with below in lowercase the corresponding DNA sequence. The N-terminal cysteine is shown in purple, N-terminal *Strep*-tag in orange, protein G in blue, amber codon for unnatural amino acid incorporation in red and hexahistidine tag in green.

```
     M  C  W  S  H  P  Q  F  E  K  G  T  M  T  F  K  L  I  I  N
  1  atgtgctggtcccatccgcagttcgagaaaggtaccatgacatttaaactgataatcaac  60

     G  K  T  L  K  G  E  I  T  I  E  A  V  D  A  *  E  A  E  K
 61  ggcaaaaccttaaaaggggagatcacaattgaggcagtcgatgcctaggaagccgagaaa  120

     I  F  K  Q  Y  A  N  D  Y  G  I  D  G  E  W  T  Y  D  D  A
121  atctttaaacaatatgctaatgattatggtattgacggagaatggacgtatgacgatgcg  180

     T  K  T  F  T  V  T  E  E  F  T  S  G  G  S  G  D  D  H  H
181  acaaaaactttcaccgtaactgaggaattcactagtggtggaagtggggacgatcatcat  240

     H  H  H  H  *
241  catcatcatcattaa  255
```

Synthesis and purification of protein G-oligonucleotide conjugates (pG-ODN)

The amino-functionalized anti-handle oligonucleotide (ODN, see Table S2) was obtained desalted from Integrated DNA Technologies and dissolved in DNase/RNase-free water (Invitrogen) at a concentration of 1 mM. In a typical reaction, 10 µL of ODN stock was added to 30 µL of 1x PBS (100 mM sodium phosphate, 150 mM NaCl, pH7.2) and 40 µL of a 2.5 mM Sulfo-SMCC (Thermo Scientific) solution in dry DMSO, and incubated at 850 rpm for 2 h at room temperature. To remove excess Sulfo-SMCC, ethanol precipitation was performed by adding 10% (v/v) 5 M NaCl and 300% (v/v) ice-cold ethanol to the reaction mixture and incubating for 75 min at -30°C. After centrifugation (19,000 g for 30 min at 4°C) the supernatant was removed and the pellet was reconstituted in 1x PBS. The precipitation was repeated once and after centrifugation the pellet was washed with 95% ice-cold ethanol (v/v, in water). The mixture was centrifuged (19,000 g for 15 min at 4°C) and the supernatant was removed. The pellet was lyophilized and stored at -30°C. The reaction was monitored with denaturing urea-PAGE (Fig. S2). In short, gels were prepared at 20% monomer concentration using the manufacturer's protocol (UreaGel, National Diagnostics) and pre-run in 1x TBE (89 mM Tris-HCl, 89 mM boric acid, 2 mM EDTA, pH 8.0) for 60 min at 150 V. Samples were diluted to a final concentration of 10 µM using TBE-urea sample buffer (Bio-Rad) and denatured at 95°C for 5 min. The gel was run for 90 min at 65 V and post-stained with SYBR Safe (Thermo Scientific). GeneRuler Ultra Low Range DNA ladder (Thermo Scientific) was included as a reference.

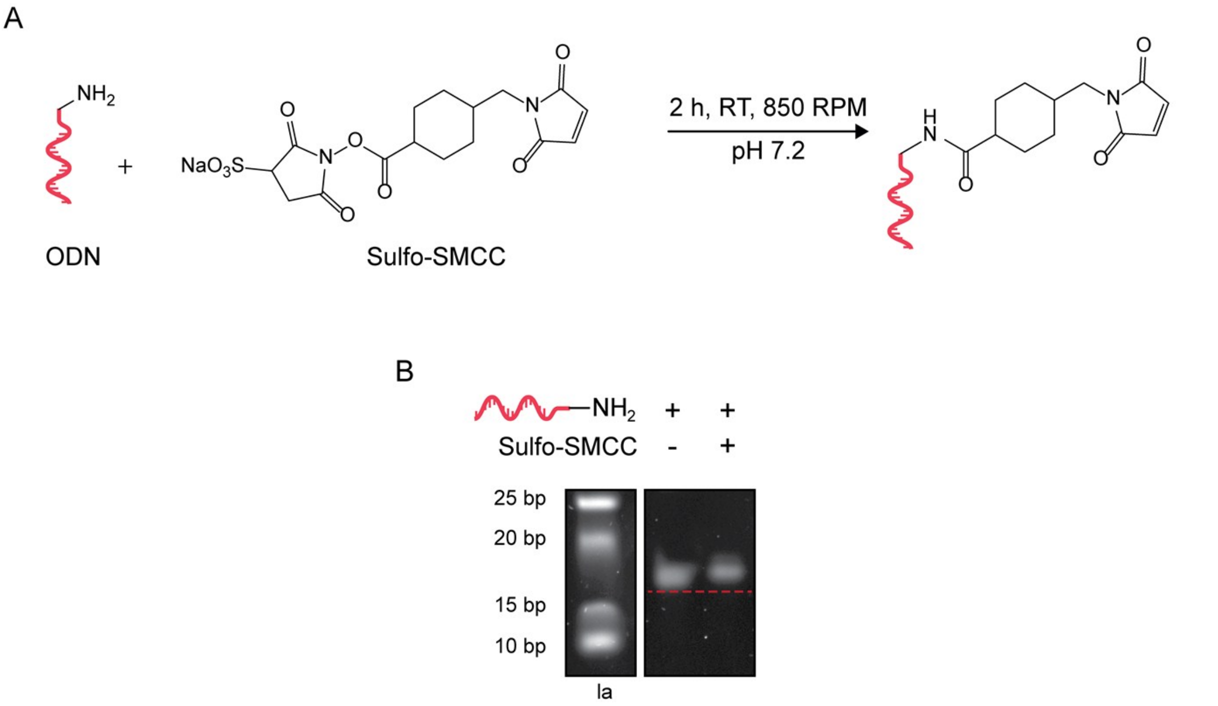

Figure S2 (a) Reaction scheme of maleimide functionalization of amino-ODN using Sulfo-SMCC. (b) 20% urea-PAGE gel analysis of the SMCC coupling shows a gel shift after reaction indicating successful SMCC coupling (calculated mass before and after reaction 6,368 Da and 6,590 Da, respectively). Labels: la, ladder; bp, base pairs.

For conjugation of pG to the SMCC-functionalized ODN, pG aliquots were first buffer exchanged to reaction buffer (100 mM sodium phosphate buffer, pH 7.0) using PD10 desalting columns (GE Healthcare). The reaction was performed by incubating pG at a final concentration of 19 µM with a 1.3 excess of maleimide-functionalized ODN in reaction buffer at a total volume of 3.2 mL. The reaction mixture was incubated at 4°C for 14 h with continuous shaking at 850 rpm. For purification, fast protein liquid chromatography (FPLC, ÄKTA Prime, GE Healthcare) was used with an anion-exchange HiTrap Q HP column (1 mL, GE Healthcare). The column was equilibrated with equilibration buffer (50 mM Tris-HCl, pH 7.5) and, after five-fold dilution in equilibration buffer, the conjugation reaction mixture was applied manually to the column. After re-equilibration with equilibration buffer supplemented with 100 mM NaCl, a salt gradient was applied with a start and end concentration of 100 and 500 mM NaCl, respectively, and a flow rate of 1 mL/min. Elution fractions of 0.5 mL were collected and analyzed by measuring on-line absorption at 280 nm and SDS-PAGE (Fig. S3).

Subsequently, Ni^{2+}-affinity chromatography was used to remove unconjugated ODN. Elution fractions of FPLC purification were pooled and loaded on a Ni-charged column (His-Bind® Resin, Novagen) and washed with wash buffer (1x PBS, 870 mM NaCl, 10% (v/v) glycerol, 20 mM imidazole, pH 7.4). The pG-ODN conjugate was eluted with elution buffer (1x PBS, 870 mM NaCl, 10% (v/v) glycerol, 250 mM imidazole, pH 7.4). The elution fractions were pooled and buffer exchanged (10 mM Tris-HCl, 1 mM EDTA, pH 7.5) using overnight dialysis (SnakeSkin, 3.5 kDa MWCO, Thermo Scientific). The concentration of pG-ODN was determined by measuring the absorption at 280 nm (ND-1000, Thermo Scientific) and comparing the value to a reference curve of equimolar mixtures of pG and ODN. Finally, the conjugates were snap frozen in liquid nitrogen and stored in 5 µL aliquots of 5 µM at -80°C in a storage buffer containing 10 mM Tris-HCl, 25 mM NaCl, 1 mM EDTA at pH 7.5.

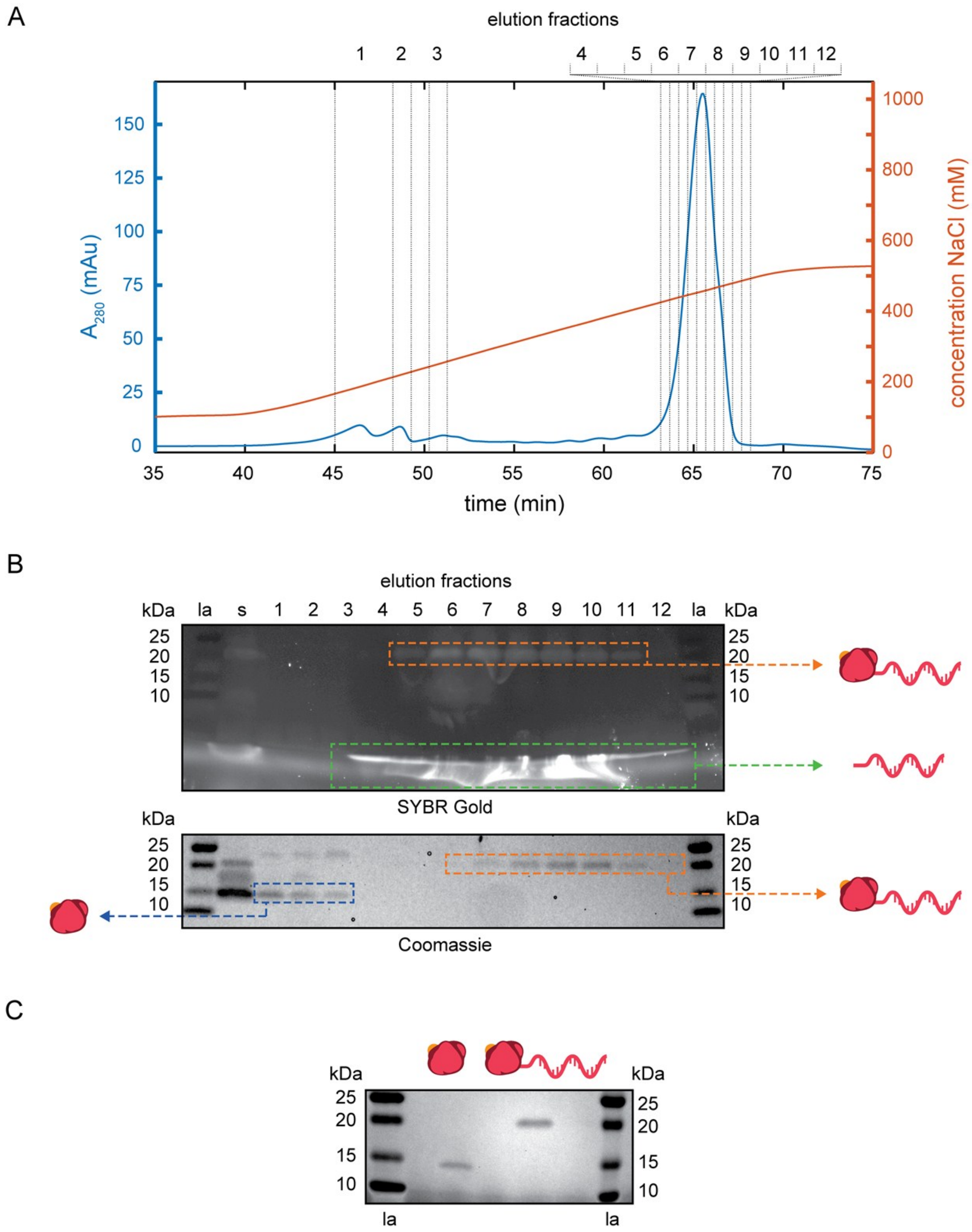

Figure S3 Purification of pG-ODN conjugates. (a) Fast protein liquid chromatography (FPLC) elution trace monitored by on-line absorption at 280 nm. (b) SDS-PAGE analysis of selected elution fractions, as indicated in (a). Labels: la, ladder; s, crude reaction mixture. The gel was first stained with SYBR Gold (top) and subsequently with Coomassie Blue (bottom). (c) SDS-PAGE gel after anion-exchange chromatography and Ni^{2+}-affinity chromatography, showing pG at an apparent mass of ~14 kDa and pure pG-ODN conjugate at ~20 kDa (calculated masses of 9.6 and 16.2 kDa, respectively). Label: la, ladder.

Design of the DNA origami rectangle

The DNA origami rectangle used in this study was designed using caDNAno v0.2 based on the *tall rectangle* design by Rothemund (Fig. S4).[3] The M13mp18 scaffold strand folds into a single-layer structure of 32 helices using 192 staple strands (Table S3). For protein incorporation, staple 59 and 134 were functionalized at the 5' end with 20-nt handle strands (handle-1, Table S2). For fluorophore incorporation for flow cytometry, staples 26, 28, 170 and 172 were functionalized at the 3' end with 20-nt handle strands (handle-2, Table S2). To correct for global twist of the DNA origami rectangle 3 base pair deletions per helix were introduced.[4] To prevent DNA origami aggregation through blunt-end stacking all 32 edge

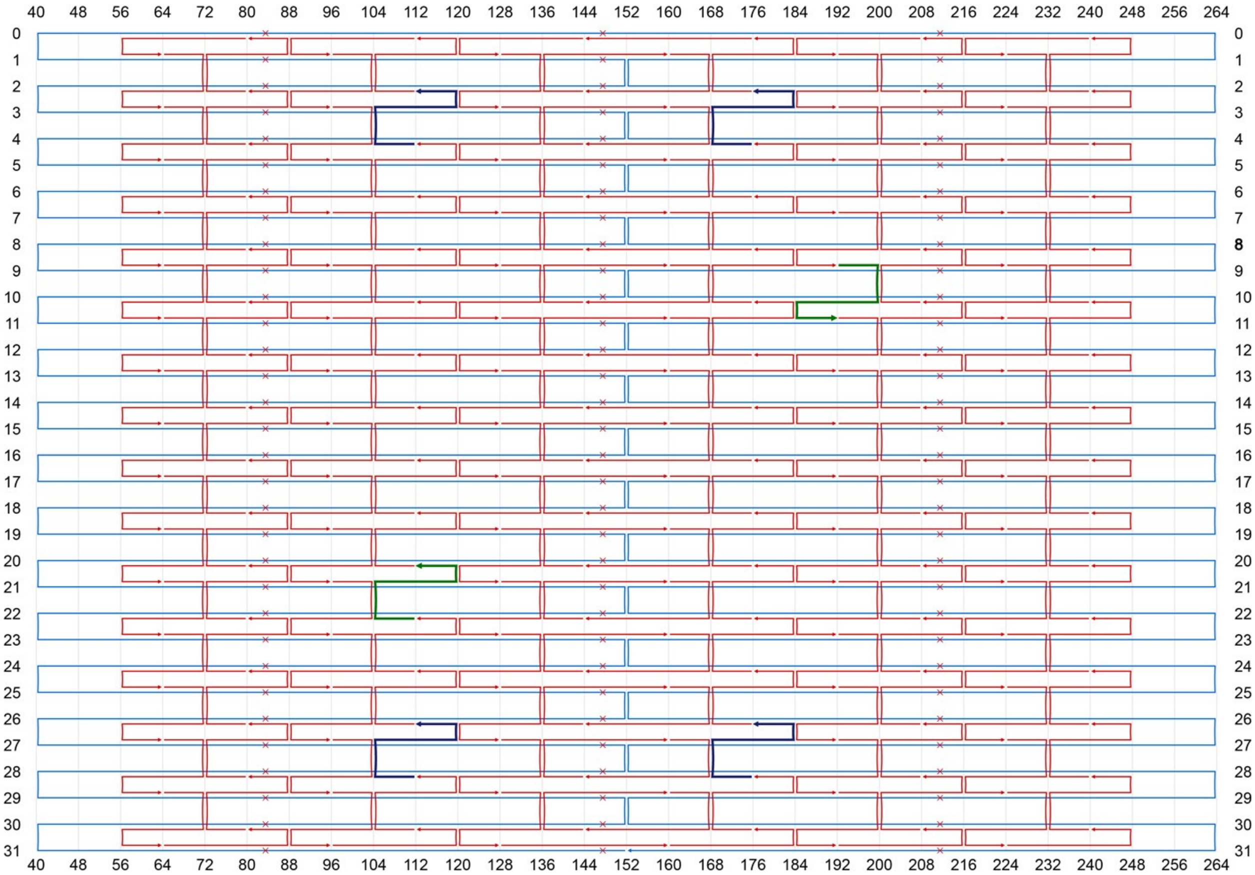

Figure S4 Schematic overview of the DNA origami rectangle. The scaffold strand is shown in light blue and unmodified staple strands in red. Staples that are used for handle-1 extension (protein incorporation) are shown in green, staples for handle-2 extension (fluorophore incorporation) shown in dark blue. Base-pair deletions to correct for global twist of the structure are indicated by crosses and 3' ends of DNA strands are indicated by arrows. Numbers on left and right indicate the reference helix number, while numbers on top and bottom indicate reference nucleotide position.

staples were omitted during folding and not listed here.

Folding and purification of DNA origami nanostructures

The 7,249 nt single-stranded scaffold strand was produced as described in literature.[5] In short, M13mp18 phage recombinant form I double-stranded DNA (New England Biolabs) was transformed in *E. coli* XL-1 Blue competent cells (Agilent) and grown overnight at 37°C on agar plates supplemented with tetracycline (10 µg/mL, Sigma Aldrich), β-D-1-thiogalactopyranoside (IPTG, 240 µg/mL, Applichem), and 5-bromo-4-chloro-3-indolyl-β-D-galactopyranoside (X-gal, 200 µg/mL, Serva), according to the manufacturer's protocol. A single well-isolated blue plaque was used to inoculate 300 mL 2xYT medium (16 g/L peptone, 5 g/L NaCl, 10 g/L yeast extract) and the culture was incubated for 5 h at 37°C. The cells were pelleted by centrifugation and the bacteriophages were extracted from the supernatant by PEG fractionation. After centrifugation the pellet was reconstituted in TE buffer (10 mM Tris, 1 mM EDTA, pH 8.5) and lysed as described[5] using buffers P2 and P3 (Qiagen). After ethanol precipitation, the single-stranded phage DNA was reconstituted in TE buffer and stored at -30°C in DNA LoBind tubes (Eppendorf). The concentration was determined by measuring the absorption at 260 nm (ND-1000, Thermo Scientific), assuming A_{260}=1 at 37.5 µg/mL. Total yield was ~6.5 mg/L culture medium.

Desalted unmodified staple strands and HPLC-purified handle-extended staple strands (see Table S3) were obtained from Integrated DNA Technologies and dissolved at a stock concentration of 500 µM in DNase/RNase-free water (Invitrogen). Folding reactions were performed at a volume of 50 µL in folding buffer (10 mM Tris, 1 mM EDTA, 10 mM $MgCl_2$, 50 mM NaCl, pH 8.0), with 25 nM scaffold strand and 250 nM of each staple strand. The reaction mixture was heated to 95°C for 15 min and then slowly cooled to 20°C at a rate of 1°C/min. Excess staple strands were removed using 100 kDa MWCO 0.5 mL Amicon centrifugal filters (Merck Millipore). Briefly, a filter was pre-wetted with 500 µL reaction buffer (10 mM Tris, 1 mM EDTA, 10 mM $MgCl_2$, 100 mM NaCl, pH 8.0). The folding mixture was diluted to 500 µL with reaction buffer, added to the filter and centrifuged at 4°C for 5 min at 5,000 g. This step was repeated for a total of three washing steps. The concentrate was recovered by inverting the filter and spinning for 2 min at 1,000 g. Samples were stored in DNA LoBind tubes at 4°C for next day use or at -30°C. The DNA origami concentration was determined by measuring the absorption at 260 nm, assuming an extinction coefficient of 1.24×10^8 M^{-1} cm^{-1}.[6,7]

Agarose gel electrophoresis was used for DNA origami folding analysis. In short, 1.5% agarose gels were cast in gel buffer (1x TAE, 10 mM $MgCl_2$, pH 8.0) supplemented with SYBR Safe. Gels were run in gel buffer for 90 min at 65 V in an ice bath. DNA origami samples were diluted just before loading to a final concentration of 4-5 nM in agarose gel loading buffer (30% (v/v) glycerol, 0.025% (w/v) bromophenol blue, 0.025% (w/v) xylene cyanol, in water). Gels were imaged using an ImageQuant 400 Digital Imager (GE Healthcare) and analyzed with ImageJ.

Photoconjugation of cetuximab and incorporation onto DNA nanostructures

Conjugation reactions for SDS-PAGE gel analysis were performed using a 5-fold excess of pG or pG-ODN, as described (Fig. 2a). For incorporation of cetuximab onto DNA nanostructures, a 5-fold excess of cetuximab was used to ensure full conversion of pG-ODN. In short, conjugation reactions were performed on a 20 µL scale in reaction buffer (10 mM Tris, 1 mM EDTA, 100 mM NaCl, pH 7.5), with 1 µM of pG-ODN and 5 µM cetuximab (Erbitux, Merck) at 4°C for 2 h under UV light (λ=365 nm, Promed UVL-36). For DNA origami hybridization, 100 nM of this reaction mixture (based on pG-ODN, assuming 100% photoconjugation) was added to a 10 nM purified DNA origami solution in reaction buffer (50 µL), and incubated at 4°C for 2 h with continuous shaking at 300 rpm.

PEG precipitation was performed by mixing 50 µL of the hybridization mixture with 50 µL of precipitation buffer (5 mM Tris, 1 mM EDTA, 505 mM NaCl, pH 8.0) supplemented with 15% (w/v) PEG-8000 (Molecular Dimensions) and incubating at 4°C for 10 min. After centrifugation at 4°C for 20 min at 5,000 g, the supernatant was carefully removed using a pipette. The pellet was re-dissolved in the initial volume of reaction buffer and equilibrated at 4°C for 30 min. The precipitation cycle was then repeated once, and the purified functionalized DNA nanostructures were stored on ice for further processing (see Fig. S6).

A431 cell culturing and analysis using flow cytometry

For flow cytometry experiments, the design of the DNA origami structures was changed to include only one incorporation site for cetuximab and four fluorophores, by exchanging unmodified staple strands for corresponding staples extended with handle-1 and handle-2, respectively (see Tables S2 and S3). Additionally, 2 µM of Cy3-labeled anti-handle-2 was added to the reaction mixture. Folding and purification was performed as described.

For fluorophore labeling, cetuximab was buffer exchanged to 100 mM sodium phosphate buffer (pH 7.0) by gel filtration using a PD10 desalting column. To this, Cyanine3 (Cy3) NHS ester (Lumiprobe) was added in a 20-fold molar excess and reacted for 2 h at room temperature. Subsequently, non-reacted dye was removed by gel filtration using a PD10 desalting column. The labeling efficiency was determined to be 4.1 Cy3 labels per antibody, based on the absorbance at 280 nm and 550 nm and assuming extinction coefficients of 210,000 M^{-1} cm^{-1} and 162,000 M^{-1} cm^{-1} for the antibody and Cy3, respectively.

Human A431 carcinoma cells overexpressing EGFR were cultured in RPMI-1640 medium (Gibco) supplemented with 10% fetal bovine serum (FBS, Gibco) and 1% penicillin/streptomycin (pen/strep, Gibco) at 37°C and 5% CO_2. The cells were passed 15 times and grown in a 175 cm^2 flask. At a confluency of ~80% the cells were harvested by incubating them in 2.5 mL trypsin for 3 min at 37°C. Subsequently the trypsin was inactivated by adding 7.5 mL medium supplemented with 10% FBS and 1% pen/strep, after which the cells were pelleted by centrifugation at 100 g. After removing the supernatant by aspiration the cells were resuspended in 10 mL 1x PBS supplemented with 0.1% (w/v) bovine serum albumin (PBS-BSA, pH 7.5) and again pelleted by centrifugation. Finally, the pelleted cells were resuspended in PBS-BSA to a stock concentration of 3.5×10^6 cells/mL.

Cetuximab incorporation and purification of functionalized DNA nanostructures was performed as described. A series of 10x cetuximab-Cy3 and DNA origami stock solutions were prepared by two-fold serial dilution in PBS-BSA. Subsequently, 25 µL of the stock solutions was mixed with 12.5 µL of the cell stock solution and 212.5 µL PBS-BSA, yielding final concentrations of 112 nM to 14 pM for cetuximab-Cy3 and 5 nM to 20 pM for DNA origami, and 175,000 cells/mL. Note that due to the small scale of the DNA origami reactions, measurements at concentrations exceeding 5 nM were not possible. After incubating the reaction mixtures for 30 min at room temperature with continuous shaking at 400 rpm the cells were pelleted by centrifugation for 5 min at 1,500 g and the supernatant was removed by aspiration. Individual samples were resuspended in PBS-BSA directly prior to measurements. Flow cytometry of the A431 cells was performed on a FACS Aria III (BD Biosciences) equipped with a 70 µm nozzle. Events representing single cells were gated based on the forward scatter versus side scatter. The fluorescence intensity was measured by excitation with a 488 nm laser and detected with a 585±7.5 nm bandpass filter. For each measurement, fluorescence intensities of 5,000 individual cells were processed and analyzed using custom-written MATLAB scripts.

To evaluate the binding strength of the interaction between cetuximab and EGFR, titration curves were constructed by taking the mean of the intensity distribution of each measurement and fitting the data to

$$fluorescence\ intensity = a + (b - a)\frac{[cetuximab]^n}{[cetuximab]^n + K_{\mathrm{D,app}}}$$

a standard noncooperative Hill equation,

where n = 1, and a and b are the fluorescence intensity values at [*cetuximab*] = 0 nM and [*cetuximab*] >> 100 nM, respectively. Apparent dissociation constants ($K_{\mathrm{D,app}}$) for cetuximab and cetuximab-functionalized DNA nanostructures were extracted and determined to be in the same range (1.9±0.2 nM and 1.2±0.2 nM, respectively). Although we were limited by the amount of DNA origami material and therefore saturating cetuximab binding was not observed, the fitting algorithm was able to estimate the $K_{\mathrm{D,app}}$. Generally, a reliable estimation of the $K_{\mathrm{D,app}}$ with a small error is possible when the binding curve has passed its inflection point. Since the calculated error on $K_{\mathrm{D,app}}$ is small and similar in both samples, we infer that the inflection point has been reached and that the fitting estimation is valid.

We also note that the absolute fluorescence intensity of the DNA origami samples is lower than for the cetuximab control (Fig. S5). Binding of cetuximab to EGFR leads to internalization and accumulation of the complex inside the cell,[8] and in the case of Cy3-labeled cetuximab, leads to intracellular accumulation of fluorophores. The presence of a large DNA origami rectangle can hamper cetuximab internalization or lead to separation between cetuximab and the fluorescently-labeled DNA origami rectangles. Therefore, we speculate that binding of cetuximab to EGFR occurs normally in the presence of DNA nanostructures, but diminished internalization and dissociation of the DNA nanostructures leads to an overall decrease in single-cell fluorescence intensity. This hypothesis is consistent with the flow cytometry titration data,

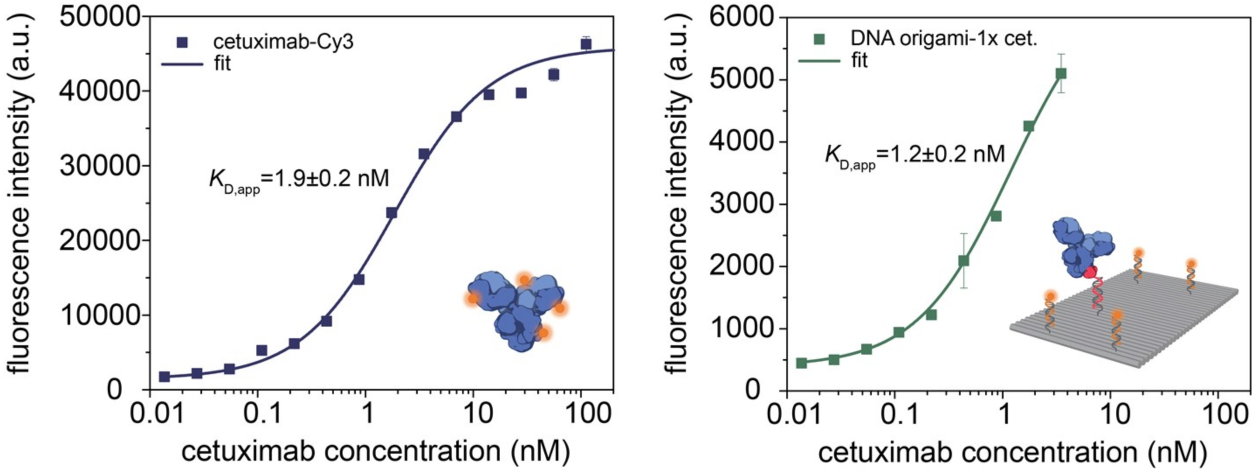

Figure S5 Flow cytometry analysis of cetuximab and cetuximab-functionalized DNA nanostructure titrations to EGFR-overexpressing cells. Serial dilutions of either Cy3-labeled cetuximab (left) or Cy3-labeled DNA origami carrying one cetuximab (right) were added to A431 cells and incubated for 30 min at room temperature. Cy3 fluorescence intensity was recorded for 5,000 cells per measurement and the mean of the intensity distribution plotted. Each data point was measured in duplo, and error bars indicate 1 s.e.m. The cetuximab concentration on the x-axis in the right plot was corrected for an incorporation efficiency of 70%, *e.g.* a DNA origami concentration of 1 nM corresponds to a cetuximab concentration of 0.7 nM. Dissociation constants were extracted by fitting the data points to the Hill equation and were determined to be in the same range for both samples (1.9±0.2 nM and 1.2±0.2 nM, respectively).

which indicates that the binding strength of cetuximab to EGFR is similar in the absence and presence of the DNA origami rectangles, even though the absolute fluorescence intensity level differs.

Photoconjugation of CD40L and incorporation onto DNA nanostructures

Conjugation reactions for SDS-PAGE analysis were performed as described (Fig. S7). Conjugation reactions for DNA nanostructure functionalization were performed on a 20 µL scale in reaction buffer (10 mM Tris, 1 mM EDTA, 100 mM NaCl, 0.1% (w/v) CHAPS, pH 7.5), with 0.3 µM of pG-ODN and 1.5 µM CD40L (recombinant human CD40 ligand-hIgG1-Fc, Thermo Scientific) at 4°C for 2 h under UV light (λ=365 nm, Promed UVL-36). For DNA origami hybridization, 80 nM of this reaction mixture (based on pG-ODN, assuming 100% photoconjugation) was added to an 8 nM purified DNA origami solution in reaction buffer (50 µL), and incubated at 4°C for 2 h with continuous shaking at 300 rpm.

After hybridization, functionalized DNA nanostructures were purified by gel extraction. Agarose gel electrophoresis was performed as described, and upon completion the correct DNA nanostructure band was excised from the gel. The band was cut into small pieces and loaded onto a Freeze 'N' Squeeze column (Bio-Rad). After centrifugation for 2 min at 2,000 g, the supernatant was collected and stored on ice for further processing.

AFM imaging of DNA nanostructures

Topographic images were acquired in tapping mode under liquid conditions on a MultiMode 8 atomic force microscope with a NanoScope IIIa controller (Veeco) using V-shaped Si_3N_4 cantilevers with sharpened pyramidal tip and a nominal spring constant of 0.04 N/m (OTR4, Bruker AFM Probes). Substrates were prepared by attaching laser-cut mica discs (~1 cm^2, Ted Pella) to Teflon (VWR) using epoxy glue. DNA origami solutions were diluted to 2 nM with imaging buffer (10 mM Tris, 1 mM EDTA, 10 mM $MgCl_2$, pH 8.0) and 5 µL was deposited on a freshly-cleaved mica substrate. The sample was incubated for 30 s and subsequently 50 µL of imaging buffer was added. In various regions on the mica surface 512×512 px images of 1×1 μm^2 or 1.5×1.5 μm^2 were acquired, optimizing the scanning and feedback parameters for each image. All images were analyzed using Gwyddion v2.39 software. Protein incorporation efficiency was calculated by counting the number of well-formed, intact DNA origami rectangles in three separate AFM images (104 rectangles, so 208 binding sites), and determining the number of proteins on those rectangles (142 for cetuximab, leading to an incorporation efficiency of 142/208≈68%, with 49/104≈47% functionalized with two antibodies).

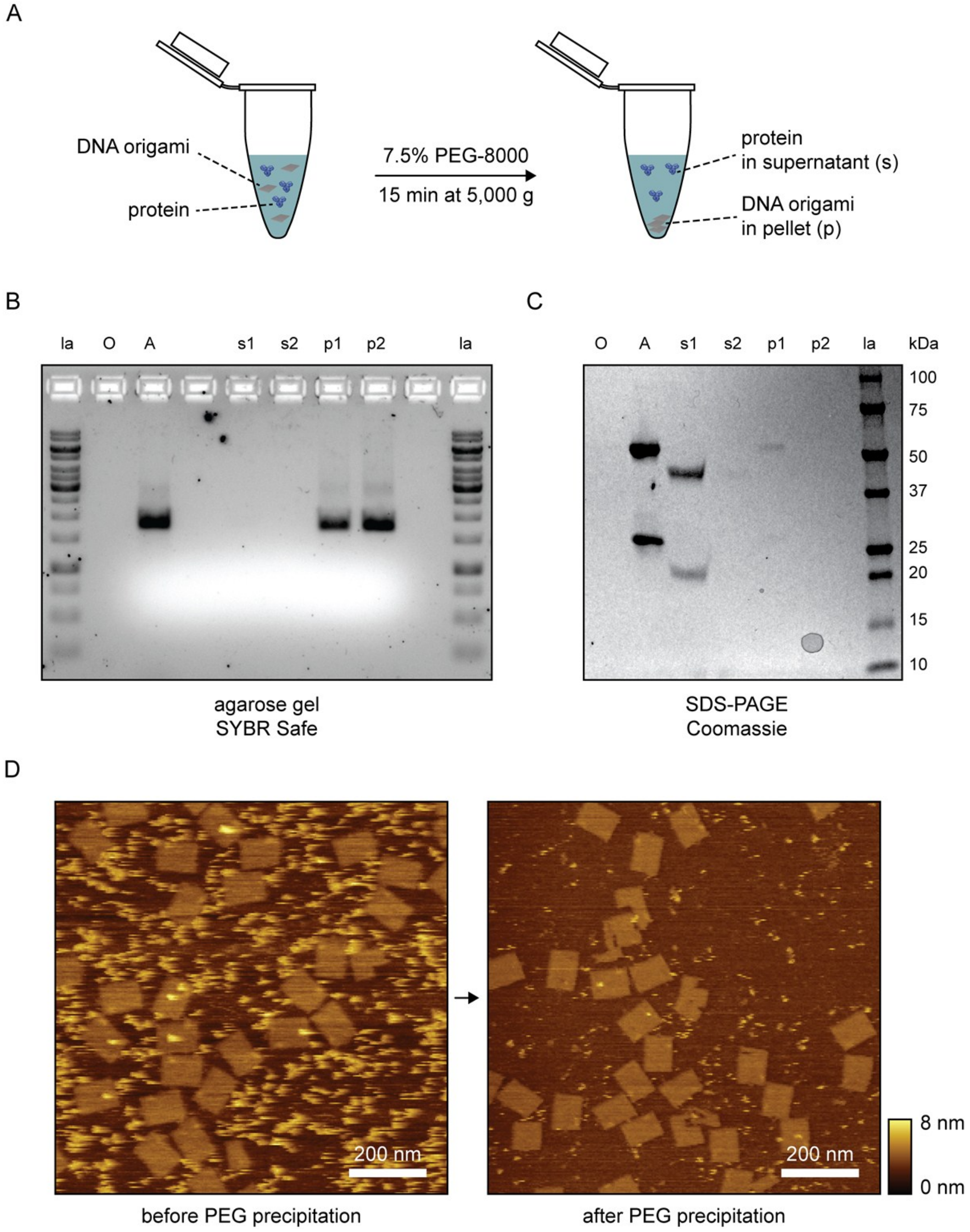

Figure S6 PEG precipitation of DNA nanostructures in the presence of unfunctionalized antibody. (a) Schematic overview of the purification principle. PEG and NaCl are added to a solution of proteins and DNA origami causing precipitation of the large negatively-charged DNA structures, while keeping the proteins in solution. After centrifugation, the supernatant containing the protein is removed and the DNA origami pellet is reconstituted in buffer. To increase purification efficiency this cycle can be repeated several times. (b-d) To show the feasibility of this strategy for purifying DNA nanostructures, a 50 µL mixture of 10 nM DNA origami and 2 µM unfunctionalized cetuximab was treated to two rounds of PEG precipitation. (b) Agarose gel analysis shows DNA origami present only in the reconstituted pellets. (c) SDS-PAGE gel analysis shows cetuximab only present in the supernatant, indicating successful removal from solution. Interaction between SDS and PEG is known to influence migration of proteins in SDS-PAGE, explaining the difference in migration between samples A and s1. Labels: la, ladder; O, reference sample of DNA origami only; A, reference sample of cetuximab only; s1, s2, p1 and p2, supernatant and pellet after first and second precipitation round, respectively. (d) AFM height images of the DNA origami-cetuximab mixture before (left) and after (right, sample p2) two rounds of PEG precipitation, proving that cetuximab was removed from the solution and that the DNA nanostructures were still intact.

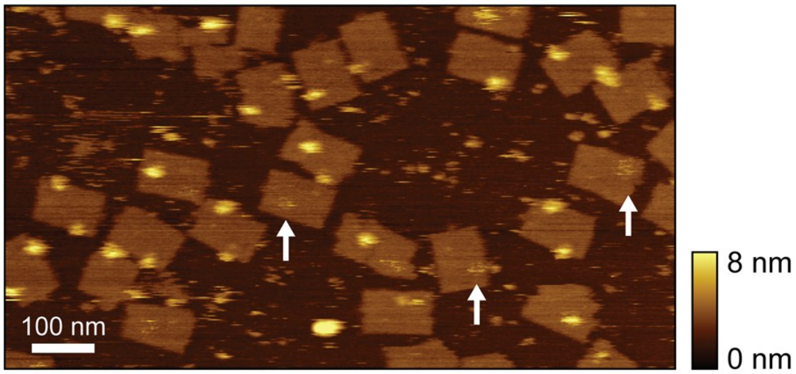

Figure S7 AFM height image of cetuximab-functionalized DNA origami structures after two rounds of PEG precipitation, prepared as described. Occasionally, deformation or displacement of cetuximab on DNA origami was observed, most likely caused by interactions of the oscillating AFM tip with the soft, flexible antibody (examples indicated by white arrows). Such artifacts can lead to an underestimation of the actual protein incorporation efficiency, and can be minimized by changing electrolyte composition of the buffer and careful tuning of AFM feedback parameters.[10]

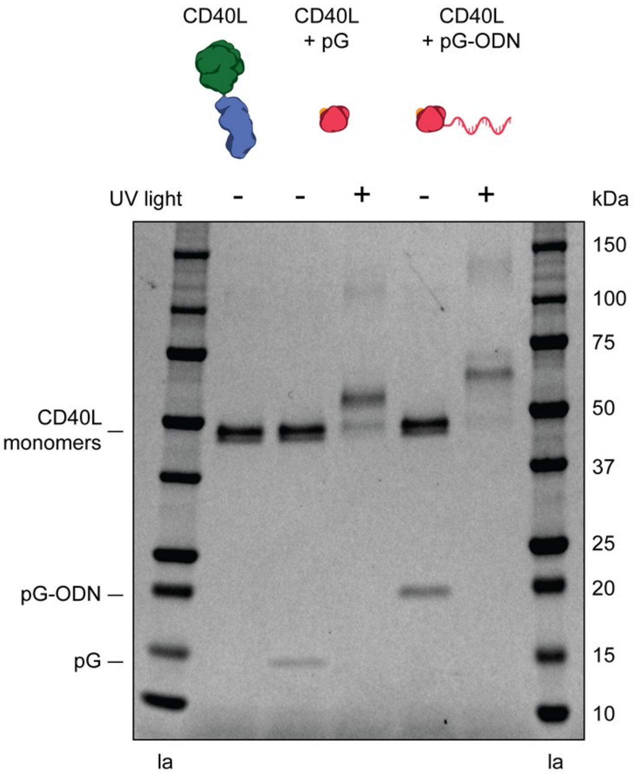

Figure S8 Characterization of CD40L-ODN conjugation using the protein G adapter with SDS-PAGE analysis. The soluble Fc-fusion protein CD40L is a disulfide-bridged homodimer with monomer mass of 43 kDa, and an apparent mass of ~45 kDa on gel due to glycosylation. Conjugation reactions were performed in 10 mM Tris, 1 mM EDTA, 100 mM NaCl, pH 7.5, with 0.4 µM of CD40L and a 5-fold molar excess of protein G (pG, 9.5 kDa) or pG-ODN (16 kDa) for 2 h at 4°C in the absence or presence of UV light (λ=365 nm). The conjugation efficiency was determined to be >90% for both pG and pG-ODN by comparing gel band intensities.

Table S1: mutagenesis primers

Name	Sequence (5' to 3')
Liu-FW	GACGATCATCATCATCATCATCATTAAGGCAGCATGGTATTCACATTGGAAGATTTC
Liu-RV	TTAATGATGATGATGATGATGATCGTCCCCACTTCCACCACTAGTGAATTCCTCAG
G2C	GAAGGAGATATAACATGTGCTGGTCCCATCCGC

Table S2: handle and anti-handle sequences

Name	Sequence (5' to 3')[a]	Length (nt)
handle-1	TCATACGACTCACTCTAGGGT	21
non-compl. handle-1	CAGTCAGTCAGTCAGTCAGTT	21
handle-2	ACTGACTGACTGACTGACTG	20
anti-handle-1	CCCTAGAGTGAGTCGTATGA-NH_2	20
anti-handle-2	Cy3-CAGTCAGTCAGTCAGTCAGT	20

[a]Underlined is a single thymine nucleotide, added as a spacer between handle and staple.

Table S3: sequences of unmodified staple strands

Staple no.	Location of 5' end[a]	Sequence (5’ to 3’)
1	0[79]	ACTGAGTTTCGTCACCAGTACAAATCATAGTT
2	0[111]	GCAAGCCCAATAGGAACCCATGTACGTCTTTC
3	0[143]	CCTCAGAGCCACCACCCTCATTTTGTATGGGA
4	0[175]	CCCTCAGAACCGCCACCCTCAGAACCGCCAC
5	0[207]	TATCACCGTACTCAGGAGGTTTAGATTATTCT
6	0[239]	GGGTTGATATAAGTATAGCCCGGATGAGACTC
7	1[64]	AGCGTAACAAAAGGCTCCAAAAGGTTCGAGGT
8	1[96]	CAGACGTTAATAATTTTTTCACGTCGATAGTT
9	1[128]	TTTTGCTAAATAGAAAGGAACAACGCCCACGC
10	1[160]	TGCCCCCTAACAGTGCCCGTATAATTTCAGC
11	1[192]	GAAACATGTAATAAGTTTTAACGGAGGTTGAG
12	1[224]	CTCAAGAGCATGGCTTTTGATGATTATTCACA
13	2[79]	CTCCAAAAGATCTAAAGTTTTGTCCGTAAC
14	2[111]	TTGCGAATAGTAAATGAATTTTCTCAGGGATA
15	2[143]	GGAGTGAGAACAACTTTCAACAGACAGTTAA
16	2[175]	CCTTGAGTGCCTATTTCGGAACCTTACCGCCA
17	2[207]	TGTACTGGAAAGTATTAAGAGGCATAGGTG
18	2[239]	GCGTCATAAAGGATTAGGATTAGCCGTCGAGA
19	3[64]	GAATTTCTCAACGGCTACAGAGGCTTCCATTA
20	3[96]	GCGCCGACGCAGCGAAAGACAGCACTACGAAG
21	3[128]	ATAACCGATAAAGGCCGCTTTTGCAAAAGAAT
22	3[160]	AGAGCCGCAGAGCCGCCACCAGAAAGGCTTG
23	3[192]	GCAGGTCATCAGAACCGCCACCCTTTTGCCTT
24	3[224]	AACAAATAACCGGAACCGCCTCCCTCATCGGC
25	4[79]	GAGGGTAGTAAACAGCTTGATACTGAAAAT
26	4[111]	CACCCTCAAATGACAACAACCATCTAAAGGAA
27	4[143]	CAGGGAGTTATATTCGGTCGCTGCCACCACC
28	4[175]	CCACCCTCCGCCAGCATTGACAGGGGTCAGTG
29	4[207]	CGCCACCCGACGATTGGCCTTGAACAGGAG

30	4[239]	GAGCCACCAATCCTCATTAAAGCCTCCAGTAA
31	5[64]	AACGGGTACGACCTGCTCCATGTTATAAGGGA
32	5[96]	GCACCAACATCATCGCCTGATAAAAGGACAGA
33	5[128]	ACACTAAACAAGCGCGAAACAAAGTAGGCTGG
34	5[160]	GTAATCAGAATGAAACCATCGATACCCCAGC
35	5[192]	TAGCGTCACCATTACCATTAGCAAAGCGCCAA
36	5[224]	ATTTTCGGGGAATTAGAGCCAGCAGATTGAGG
37	6[79]	GAAATCCGAAATACGTAATGCCATCGGAAC
38	6[111]	AGATTTGTCTAAAACGAAAGAGGCGGGATCGT
39	6[143]	GATTATACACACTCATCTTTGACGCAGCACC
40	6[175]	ACGTCACCTAGCGACAGAATCAAGCAGAGCCA
41	6[207]	CAGTAGCAGACTGTAGCGCGTTTTCAGAGC
42	6[239]	GCCATTTGTCATAGCCCCCTTATTCGGAACCA
43	7[64]	ACCGAACTAAACACCAGAACGAGTCTTTAATC
44	7[96]	TGAACGGTTCATTCAGTGAATAAGTTAAGAAC
45	7[128]	CTGACCTTATTCATTACCCAAATCTTGGGAAG
46	7[160]	AATAGAAAGAATAAGTTTATTTTGTGACAAG
47	7[192]	AGACAAAAGCAACATATAAAAGAAAAGTAAGC
48	7[224]	GAGGGAAGAGCAAACGTAGAAAATAAGGAAAC
49	8[79]	CTGACGAGGACCAACTTTGAAAGTTGTGTC
50	8[111]	AAAGCTGCGTACAGACCAGGCGCATACAACGG
51	8[143]	AACCGGATCATCAAGAGTAATCTTCACAATC
52	8[175]	ACACCACGATTCATATGGTTTACCGGCCGGAA
53	8[207]	TAAAGGTGGGGCGACATTCAACCAAATCAC
54	8[239]	AGTATGTTGTAAATATTGACGGAACGACTTGA
55	9[64]	ATTGTGAAACATAACGCCAAAAGGTAACCCTC
56	9[96]	TGGCTCATTTTAGGAATACCACATCAAAATAG
57	9[128]	AAAAATCTTTATTACAGGTAGAAATTGCCAGA
58	9[160]	TCTTACCGGAAACAATGAAATAGCACTAACG
**59**	**9[192]**	**AGATAGCCAAGAATTGAGTTAAGCGTTTAACG**
60	9[224]	CGAGGAAAATTGAGCGCTAATATCTACAGAGA
61	10[79]	ATGCAGATTTACCTTATGCGATTGCTTGCC
62	10[111]	AGTTGAGATATACCAGTCAGGACGAACGTAAC
63	10[143]	GAACAACAACGTTAATAAAACGAAATAGCTA
64	10[175]	AAGAGCAAAAGCCCTTTTTAAGAAACGCAAAG
65	10[207]	TAACCCACGAACAAAGTTACCAGACATACA
66	10[239]	AGAGGGTACGCAATAATAACGGAATATTACGC
67	11[64]	GTTTACCAACGAGAATGACCATAAAGTCAGAA
68	11[96]	CGAGAGGCTCCCCCTCAAATGCTTATTAAGAG
69	11[128]	GGGGGTAAATACTGCGGAATCGTCGCGTTTTA
70	11[160]	AATCCAAAATAAACAGCCATATTAACTGGAT
71	11[192]	TCAAAAATGTCTTTCCAGAGCCTAAGGCTTAT
72	11[224]	GAATAACATTTATCCTGAATCTTAGTTTTAGC
73	12[79]	TTCAGAAAGACGACGATAAAAACTCAACTA
74	12[111]	TCATTGAATTTTGCAAAAGAAGTTGATTCATC
75	12[143]	AGCGTCCATAGTAAAATGTTTAGTTTATCCC
76	12[175]	GTTACAAATAAGAAACGATTTTTTCCAATAAT
77	12[207]	TAACGAGCGAAAATAGCAGCCTTAGAGAGA
78	12[239]	GCTACAATTAAAAACAGGGAAGCGACAAAGTC
79	13[64]	GCAAAGCGTGGCTTAGAGCTTAATTAAATATG
80	13[96]	GAAGCCCGGCTCCTTTTGATAAGAGTTTCATT
81	13[128]	ATTCGAGCCAACAGGTCAGGATTACTGCGAAC
82	13[160]	AATAGCAAATCGTAGGAATCATTAACCGGAA
83	13[192]	CCGGTATTTCATCGAGAACAAGCAACGCGCCT
84	13[224]	GAACCTCCCCAAGAACGGGTATTATGAACAAG
85	14[79]	TTTGCGGAGATTGCATCAAAAAGTAAACAG
86	14[111]	CTTTAATTAAAGACTTCAAATATCATAAATAT

87	14[143]	GCAAACTCTTCAAAGCGAACCAGCCGCGCCC
88	14[175]	TTATTTTCGCAAATCAGATATAGAATTTGCCA
89	14[207]	TACCGCACCTAAGAACGCGAGGCCCAACGC
90	14[239]	TTATCATTCGACTTGCGGGAGGTTTGCACCCA
91	15[64]	CAACTAAACTACTAATAGTAGTAGAAAGAATT
92	15[96]	CCATATAATGGGGCGCGAGCTGAACAGAGCAT
93	15[128]	GAGTAGATTGGTCAATAACCTGTTACATTATG
94	15[160]	AACAACATAATTCTGTCCAGACGAGATACAT
95	15[192]	GTTTATCAAGAGAATATAAAGTACAAAAGCCT
96	15[224]	AAAAATAATTTAGGCAGAGGCATTAAATTCTT
97	16[79]	CATCAATTGTACGGTGTCTGGAAGGTCATT
98	16[111]	TTTTCATTCAGTTGATTCCCAATTGAGAGTAC
99	16[143]	TTCGCAAATTAGTTTGACCATTACGACAATA
100	16[175]	GGTAAAGTGTTCAGCTAATGCAGAAGCCGTTT
101	16[207]	CAGTAATAACAATAGATAAGTCCAACCAAG
102	16[239]	ACATGTAATATCCCATCCTAATTTGTCTTTCC
103	17[64]	AGCAAAATGTAAAGATTCAAAAGGCAATATGA
104	17[96]	AAAGCTAAATTTTAAATGCAATGCATTAATGC
105	17[128]	ACCCTGTACGCAAGGATAAAAATTGATCTACA
106	17[160]	AACACCGGTAAATAAGGCGTTAAAAAGCCTT
107	17[192]	GTTTAGTAAAATTTAATGGTTTGAGGTCTGAG
108	17[224]	ACCAGTATATATATTTTAGTTAATTTAGGTTG
109	18[79]	ATGTGTAGTAAGCAATAAAGCCTAAGGTGG
110	18[111]	CCTCATATATCGGTTGTACCAAAATAGCTATA
111	18[143]	TATTTCAAATACTTTTGCGGGAGTAAGAATA
112	18[175]	CCGTGTGAAATCATAATTACTAGACGACAAAA
113	18[207]	TCTGACCTTCATATGCGTTATACTTCGAGC
114	18[239]	TTTTTCAAAAAGCCAACGCTCAACCAACGCCA
115	19[64]	TATTCAACTCAGAAAAGCCCCAAATGTAAACG
116	19[96]	CGGAGAGGGCATGTCAATCATATGTTAAATTT
117	19[128]	AAGGCTATAAGAGAATCGATGAACCCAATAGG
118	19[160]	AATAGTGATAGATTAAGACGCTGAGAGTCTG
119	19[192]	AGACTACCCCCTTAGAATCCTTGAGATGAAAC
120	19[224]	GGTTATATCTTCTGTAAATCGTCGTTACATTT
121	20[79]	GTTGATAACGTTCTAGCTGATAACTGAGTA
122	20[111]	TAAAACTAGTAGCTATTTTTGAGATTTAGAAC
123	20[143]	GAGCAAACCAGGTCATTGCCTGAGAAGAGTC
124	20[175]	CGATAGCTATTTATCAAAATCATAAATACCGA
125	20[207]	TTAATTTTTTTTTAACCTCCGGCTTCATCT
126	20[239]	TAACCTTGAACTATATGTAAATGCGAGAAAAC
127	21[64]	TTAATATTACGGCGGATTGACCGTCATCGTAA
128	21[96]	TTGTTAAATAACAACCCGTCGGATGACGACGA
129	21[128]	AACGCCATGCCAGCTTTCATCAACACTCCAGC
130	21[160]	TCAATTACTCGCGCAGAGGCGAATTCTGGCC
131	21[192]	AAACATCACGCCTGATTGCTTTGAATAATGGA
132	21[224]	AACAATTTCCTTTTACATCGGGAGAATTATTT
133	22[79]	GGGAACAATTGTTAAAATTCGCATACCCCG
**134**	**22[111]**	**TGAGCGAGTCAGCTCATTTTTTAAGGTAATCG**
135	22[143]	TTCCTGTACAAAAATAATTCGCGTATTCATT
136	22[175]	TTACAAAACTGAGCAAAAGAAGATAAACATAG
137	22[207]	AACGGATTAGAAAACAAAATTAACTATTAA
138	22[239]	TAACAGTACATTTGAATTACCTTTTGAGTGAA
139	23[64]	CCGTGCATGCTATTACGCCAGCTGTTGGGTAA
140	23[96]	CAGTATCGGTTGGGAAGGGCGATCCGTTGTAA
141	23[128]	CAGCTTTCAAAGCGCCATTCGCCAATGCCTGC
142	23[160]	TGATTGTTGATGGCAATTCATCAATGCCGGA
143	23[192]	AGGGTTAGCGGAATTATCATCATAGATAATAC

144	23[224]	GCACGTAATTTTGCGGAACAAAGAACTTTACA
145	24[79]	GCCTCTTCCTGCCAGTTTGAGGGTCTCCGT
146	24[111]	GCGCAACTGCCTCAGGAAGATCGCATTAAATG
147	24[143]	AACCAGGCCGGCACCGCTTCTGGTATAATCC
148	24[175]	TATCAGATTGGATTATACTTCTGAATACCAAG
149	24[207]	AGAAGGAGAACCTACCATATCAAAAACAAT
150	24[239]	CATTATCAAACAGAAATAAAGAAAATATACAG
151	25[64]	CGCCAGGGCATACGAGCCGGAAGCGAGCTAAC
152	25[96]	AACGACGGGAAATTGTTATCCGCTCTGCCCGC
153	25[128]	AGGTCGACTTCGTAATCATGGTCACCAGCTGC
154	25[160]	ACTAATAGAAAATATCTTTAGGAGGGTACCG
155	25[192]	ATTTGAGGCAACAGTTGAAAGGAAAAAACAGA
156	25[224]	AACAATTCCCCTCAATCAATATCTCGCCTGCA
157	26[79]	CCACACAATTTTCCCAGTCACGAGGTGCGG
158	26[111]	TCCTGTGTCCAGTGCCAAGCTTGCTTCAGGCT
159	26[143]	AGCTCGAATCTAGAGGATCCCCGCACTAACA
160	26[175]	GGTTATCTATTAGAGCCGTCAATATTCCTGAT
161	26[207]	TGGCAAATATTTAGAAGTATTAGAACCACC
162	26[239]	ATATCAAAGACAACTCGTATTAAATTGAGTAA
163	27[64]	TCACATTACACCGCCTGGCCCTGACCCCAGCA
164	27[96]	TTTCCAGTCCAGTGAGACGGGCAAGTTCCGAA
165	27[128]	ATTAATGACGTATTGGGCGCCAGGAAAGAATA
166	27[160]	CCGAACGACCTAAAACATCGCCATGGGAGAG
167	27[192]	GGTGAGGCGGCTATTAGTCTTTAATCAATCGT
168	27[224]	ACAGTGCCAGAATACGTGGCACAGCAGATTCA
169	28[79]	TTGCCCTTATTGCGTTGCGCTCACACAATT
170	28[111]	TCTTTTCACGGGAAACCTGTCGTGTAGCTGTT
171	28[143]	GCGGTTTGATCGGCCAACGCGCGTAAAAATA
172	28[175]	CTGATAGCACCACCAGCAGAAGATTTGAGGAA
173	28[207]	TTTTGAATGGTCAGTATTAACACGGTCAGT
174	28[239]	AAAGCGTAACGCTGAGAGCCAGCAAACCTCAA
175	29[64]	GGCGAAAAATGGCCCACTACGTGAGGTGCCGT
176	29[96]	ATCGGCAAGTCAAAGGGCGAAAAAAGGGAGCC
177	29[128]	GCCCGAGAAGAGTCCACTATTAAAAGCCGGCG
178	29[160]	ATGGAAATGCCATTGCAACAGGAATTCCAGT
179	29[192]	CTGAAATGTGCTGGTAATATCCAGGAATCCTG
180	29[224]	CCAGTCACGCCTGAGTAGAAGAACGGCCACCG
181	30[79]	TCAGGGCGTCCTGTTTGATGGTGCAGCTGA
182	30[111]	ACTCCAACAATCCCTTATAAATCAGTGGTTTT
183	30[143]	TTGGAACATAGGGTTGAGTGTTGAAACGCTC
184	30[175]	TACCGCCAACCTACATTTTGACGCTGCGCGAA
185	30[207]	ATCGGCCTGATTATTTACATTGGACAATAT
186	30[239]	CATCACTTACGACCAGTAATAAAACTGACCTG
187	31[64]	AAAGCACTAAATCGGAACCCTAACCGTCTA
188	31[96]	CCCGATTTAGAGCTTGACGGGGAAGAACGTGG
189	31[128]	AACGTGGCGAGAAAGGAAGGGAATTAAAGGG
190	31[160]	ATTTTAGACAGGAACGGTACGCCAAACAATAT
191	31[192]	AGAAGTGTTTTTATAATCAGTGATCAAACT
192	31[224]	AGTAAAAGAGTCTGTCCATCACGCAGTAATAA

[a]Location of the 5’ end is indicated by the reference helix number used in Fig. S1, with the reference nucleotide position denoted in brackets.

Supplementary references

1 J. Z. Hui, S. Tamsen, Y. Song and A. Tsourkas, *Bioconjug. Chem.*, 2015, **26**, 1456–1460.

2 H. Liu and J. H. Naismith, *BMC Biotechnol.*, 2008, **8**, 91.

3 P. W. K. Rothemund, *Nature*, 2006, **440**, 297–302.

4 J. J. Schmied, M. Raab, C. Forthmann, E. Pibiri, B. Wünsch, T. Dammeyer and P. Tinnefeld, *Nat. Protoc.*, 2014, **9**, 1367–1391.

5 S. M. Douglas, J. J. Chou and W. M. Shih, *Proc. Natl. Acad. Sci. U. S. A.*, 2007, **104**, 6644–6648.

6 A. M. Hung, C. M. Micheel, L. D. Bozano, L. W. Osterbur, G. M. Wallraff and J. N. Cha, *Nat. Nanotechnol.*, 2010, **5**, 121–126.

7 W. A. Kibbe, *Nucleic Acids Res.*, 2007, **35**, 43–46.

8 G. Galizia, E. Lieto, F. De Vita, M. Orditura, P. Castellano, T. Troiani, V. Imperatore and F. Ciardiello, *Oncogene*, 2007, **26**, 3654–3660.

9 I. E. Gentle, D. P. De Souza and M. Baca, *Bioconjug. Chem.*, 2004, **15**, 658–663.

10 D. J. Müller, H. Janovjak, T. Lehto, L. Kuerschner and K. Anderson, *Prog. Biophys. Mol. Biol.*, 2002, **79**, 1–43.
